# Supplementary material for: The Impact of Cytomegalovirus Infection on Natural Killer and CD8+ T Cell Phenotype in Multiple Sclerosis
Source: Biology (Basel). 2024 Feb 28;13(3):154. doi: 10.3390/biology13030154 (PMC10968113; doi:10.3390/biology13030154)
Supplement: Supplementary file 1 [file biology-13-00154-s001.zip › biology-2795954-supplementary.pdf]

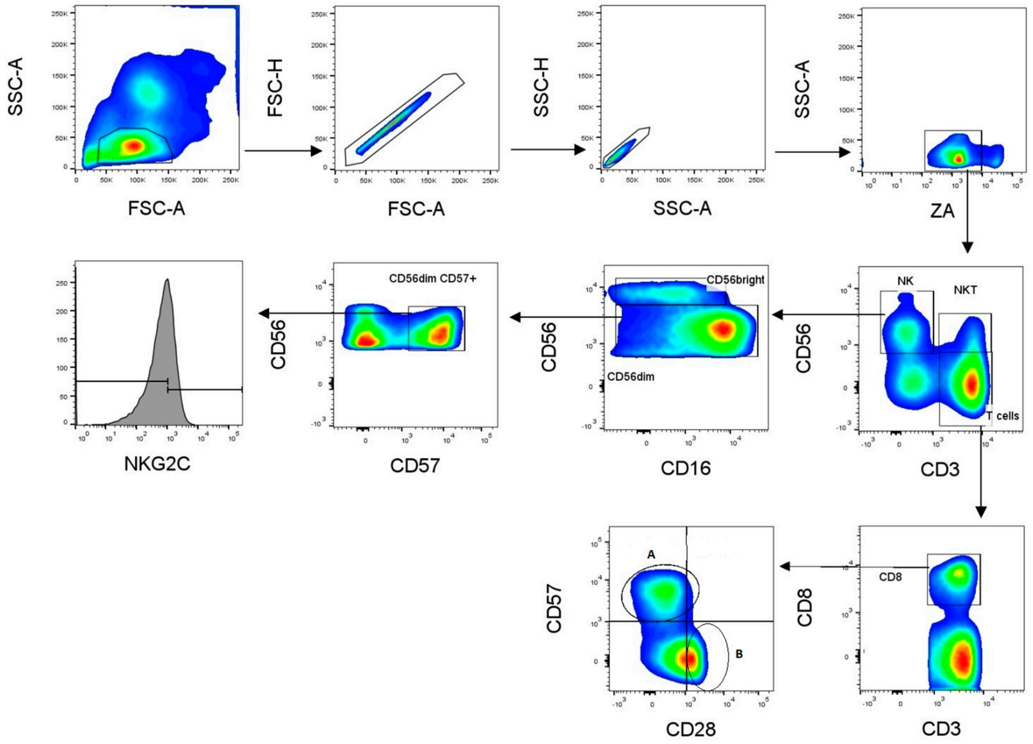

Supplementary Figure S1. Gating strategy. PBMC were gated based on forward (FSC) and side scatter (SSC), and then gated for singlets. After the exclusion of dead cells with Zombie Aqua dye, NK, T and NKT-like cells were identified based on CD3 and CD56 expression. NK cells were identified as CD3- CD56+. Based on CD16 and CD56 expression, CD56dim and CD56bright NK cells were identified. CD57+ NK cells were identified in CD56dim gate. The NKG2C median fluorescence intensity (MFI) was evaluated on CD57+ NK cells subset. NKT-like cells were identified as CD3+ CD56+. T cells were identified as CD3+ CD56-. In T-cell gate, we identified CD8+ based on CD3 and CD8 expression. In CD8 gate, we further identified (A) late-differentiated CD8 T cells and (B) early-differentiated CD8 T cells based on CD28/CD57 expression.
